# Presence of six different lesion types suggests diverse mechanisms of tissue injury in neuromyelitis optica

**DOI:** 10.1007/s00401-013-1116-7

**Published:** 2013-04-12

**Authors:** Tatsuro Misu, Romana Höftberger, Kazuo Fujihara, Isabella Wimmer, Yoshiki Takai, Shuhei Nishiyama, Ichiro Nakashima, Hidehiko Konno, Monika Bradl, Ferenc Garzuly, Yasuto Itoyama, Masashi Aoki, Hans Lassmann

**Affiliations:** 1 Department of Multiple Sclerosis Therapeutics, Tohoku University Graduate School of Medicine, Sendai, Japan; 2 Institute of Neurology, Medical University of Vienna, Vienna, Austria; 3 Department of Neuroimmunology, Center for Brain Research, Medical University of Vienna, Spitalgasse 4, 1090 Wien, Austria; 4 Department of Neurology, Tohoku University Graduate School of Medicine, Sendai, Japan; 5 Department of Neurology, National Nishitaga Hospital, Sendai, Japan; 6 Department of Pathology, Markusovszky Hospital, Szombathely, Hungary; 7 National Center Hospital NCNP, Tokyo, Japan

**Keywords:** Neuromyelitis optica, Astrocytes, Demyelination, Complement

## Abstract

Neuromyelitis optica (NMO) is an autoimmune disease targeting aquaporin 4 (AQP4), localized mainly at the astrocytic foot processes. Loss of AQP4 and glial fibrillary acidic protein (GFAP) was reported, but the pathological significance of astrocytopathy is still controversial. Here we show that active lesions in NMO display a wide spectrum of pathology even within a single tissue block of an individual patient. We have distinguished six different lesion types. The first reflects complement deposition at the surface of astrocytes, associated with granulocyte infiltration and astrocyte necrosis and followed by demyelination, global tissue destruction and the formation of cystic, necrotic lesions (lesion type 2). Such destructive lesions lead to Wallerian degeneration in lesion-related tracts (lesion type 3). Around active NMO lesions AQP4 may selectively be lost in the absence of aquaporin 1 (AQP1) loss or other structural damage (lesion type 4). Another pattern is characterized by clasmatodendrosis of astrocytes, defined by cytoplasmic swelling and vacuolation, beading and dissolution of their processes and nuclear alterations resembling apoptosis, which was associated with internalization of AQP4 and AQP1 and astrocyte apoptosis in the absence of complement activation. Such lesions give rise to extensive astrocyte loss, which may occur in part in the absence of any other tissue injury, such as demyelination or axonal degeneration (lesion type 5). Finally, lesions with a variable degree of astrocyte clasmatodendrosis are found, which show plaque-like primary demyelination that is associated with oligodendrocyte apoptosis, but with preservation of axons (lesion type 6). In active multiple sclerosis (MS) lesions astrocytes reveal changes of reactive protoplasmatic or fibrillary gliosis. Only in a subset of lesions, in patients with aggressive disease, loss of AQP4 is observed in the initial stage of their formation, which is associated with retraction of astrocyte processes in the absence of complement deposition, granulocyte infiltration or loss of AQP1 or astrocytes. Our data underline the primary assault of astrocytes in NMO lesions, but also indicate that different mechanisms of tissue injury operate in parallel in the same patient and even within the same lesion.

**Electronic supplementary material:**

The online version of this article (doi:10.1007/s00401-013-1116-7) contains supplementary material, which is available to authorized users.

## Introduction

Neuromyelitis optica (NMO) is a chronic inflammatory disease of the central nervous system, resulting in demyelinating and destructive lesions predominantly in the spinal cord and the optic system [6, 28]. Recently, auto-antibodies directed against the astrocyte water channel aquaporin 4 (AQP4) have been discovered in NMO patients, which turned out to be a highly specific and sensitive paraclinical diagnostic marker of the disease [11, 25, 26]. Furthermore, the potential pathogenicity of these auto-antibodies has been shown in in vitro and in vivo experiments [2, 3, 20, 42, 46]. From these data it is now well established that AQP4 antibodies, when getting access to the central nervous system compartment in vivo, can destroy astrocytes [8]. It is assumed that the antibodies drive complement-dependent lysis, and that granulocytes and eosinophils recruited into the lesions are major effector cells [42]. Demyelination and axonal destruction may in part be mediated by excitotoxic mechanisms, which may develop when the excitatory amino acid transporter 2 (EAAT2) is lost from dysfunctional astrocytes [13, 30]. In addition, loss of AQP4 from astrocytes may disturb water homeostasis and result in brain edema [12, 14]. However, to what extent these concepts, mainly developed in in vitro models, are also operating in the patient’s lesions in vivo, is less clear. In addition, it remains to be determined, whether similar mechanisms of tissue injury are also relevant for the development of demyelinating lesions in multiple sclerosis patients [38]. This question has gained further attention, since it has recently been described that about half of all MS patients have circulating autoantibodies against a potassium channel expressed on astrocytic foot processes (Kir 4.1), and that these antibodies may destroy astrocytes in vitro in a complement-dependent manner [50]. In this study we performed a detailed comparison of astrocyte pathology in relation to demyelination and neurodegeneration in active NMO and MS lesions. As described before [31, 35, 36, 43], our studies show that astrocyte pathology is unique and highly characteristic in NMO, but that different mechanisms lead to astrocyte destruction, demyelination and neurodegeneration. In contrast, we did not find evidence for antibody or complement-mediated astrocyte injury in MS lesions.

## Materials and methods

### Cases and material

This study was performed on paraffin-embedded, formalin-fixed archival material of active lesions from NMO patients (*n* = 7), from multiple sclerosis patients, including acute MS (*n* = 6), secondary progressive MS (*n* = 6) and primary progressive MS (*n* = 6), and non-neurological controls (*n* = 3; Table 1). The cases were selected from a much larger sample of archival autopsy material, collected in the Department of Neurology at the Tohoku University School of Medicine and the Brain Research Institute at the University of Vienna, on the basis of presence of active lesions and suitability for detailed immunohistochemical investigations. From the NMO cases, four cases fulfilled the Wingerchuck [56] clinical criteria of definite NMO (499, 216, A10-42 and 2001-50), two of a limited form of NMO (12193 and A10-36) and one case (24–81) presented with a single destructive lesion in the medulla oblongata, with NMO typical pathology [28]. Since half of the NMO patients died before tests for AQP4 antibodies were available, pathological diagnostic criteria for NMO were used, which included lesional topography, the presence of longitudinally extensive lesions in the spinal cord mainly affecting grey matter, of active lesions with marked inflammation and tissue damage in vasculocentric pattern, and/or destructive inflammatory lesions with extensive loss of astrocytes, associated with demyelination and profound axonal destruction [28]. In contrast, all MS patients showed focal inflammatory demyelinating lesions throughout the central nervous system (CNS) and a variable extent of cortical demyelination and diffuse white matter injury [22]. In addition, clinical history and results of whole body autopsy, where available, were reviewed. The study was approved by the Ethic Commission of the Medical University of Vienna (Nr. 087/01/2012) and the Tohoku University Graduate School of Medicine (No. 2009-143/2011-74).

**Table 1:** Demographics of NMO, MS and control patients

Case	Age	Gender	Dis.Dur.	AQP-4 Ab	Les. Location	Les. Type
499	20	Female	4 years	n.d.	SC, O, Brain	1, 2, 3, 4, 5, 6
216	30	Female	5 years	n.d.	SC, Med.	1, 2, 4, 5
24-81	46	Female	0.5 months	n.d.	Med.	1, 2
12193	57	Female	8 months	Positive	SC, Med.	2, 4, 5, 6
A10-42	75	Female	21 years	Positive	SC, O	4
A10-36	82	Female	10 years	Positive	SC, Brain	4, 6
2001-50	63	Female	20 years	n.d.	SC, O	1, 2, 3, 4, 5, 6
AMS 1	34	Female	4 months	n.d.	Brain	DM, p. AQP4 loss
AMS 2	35	Male	1.5 months	n.d.	SC, Med, Brain	DM, p. AQP4 loss
AMS 3	45	Male	0.2 months	n.d.	Brain	DM, p. AQP4 loss
AMS 4	45	Male	0.6 months	n.d.	Brain	DM, p. AQP4 loss
AMS 5	52	Male	1.5 months	n.d.	Brain	DM, RG
AMS 6	78	Male	2 months	n.d.	Med, Brain	DM, RG
SPMS 1	34	Male	120 months	n.d.	SC, Med, Brain	DM, RG
SPMS 2	41	Male	137 months	n.d.	Med, Brain	DM, RG
SPMS 3	46	Female	444 months	n.d.	SC, Med, Brain	DM, RG
SPMS 4	53	Female	241 months	n.d.	Brain	DM, RG
SPMS 5	56	Male	372 months	n.d.	Med, Brain	DM, RG
SPMS 6	66	Female	96 months	n.d.	Brain	DM, RG
PPMS 1	34	Male	204 months	n.d.	Med, Brain	DM, RG
PPMS 2	53	Male	168 months	n.d.	Brain	DM, RG
PPMS 3	54	Female	72 months	n.d.	Brain	DM, RG
PPMS 4	55	Female	60 months	n.d.	Brain	DM, RG
PPMS 5	67	Male	87 months	n.d.	Med, Brain	DM, RG
PPMS 6	71	Female	264 months	n.d.	Brain	DM, RG
CO1	39	Female	0	n.d.		0
CO2	45	Female	0	n.d.		0
CO3	30	Female	0	n.d.		0

*Dis.Dur* disease duration, *Les.Locat* lesion location, *Les.Type* lesional type, *AMS* acute multiple sclerosis, *SPMS* secondary progressive multiple sclerosis, *PPMS* primary progressive multiple sclerosis, *CO* controls, *SC* spinal cord, *O* optic nerve, *Med* medulla oblongata, *DM* demyelinated lesions, *p AQP4 loss* partial aquaporin 4 loss, *RG* reactive gliosis

#### Neuropathological techniques and immunohistochemistry

The brain and spinal cords were fixed with 10 % formalin and multiple tissue blocks were embedded in paraffin. Tissue slices of 4–6 μm thickness were cut and mounted serially on numbered slides. Histological examinations were performed by hematoxylin and eosin (HE), Klüver-Barrera (KB), and Bielschowsky’s silver impregnation axonal stain.

Sections were deparaffinized twice with xylol substitute (XEM) (Fluka analytical, Germany) for 20 min each, rinsed twice in 96 % ethanol, treated with hydrogen peroxide in methanol for 30 min to block endogenous peroxidases, rehydrated in a descending series of ethanol and further incubated for 1 h in phosphate buffered saline containing 10 % fetal calf serum (PBS/FCS) to block nonspecific antibody binding. Antigen retrieval was performed by heating the sections for 60–90 min in EDTA (1 mM EDTA, 10 mM Tris, pH 8.5 or 9) or 0.1 mM citrate buffer (pH 6) in a household food steamer device. The primary antibodies (Table 2) were applied overnight in PBS/FCS. Afterwards, the slides were washed 3–4 times in PBS. Then the slides were incubated with biotinylated secondary antibodies (sheep anti-mouse, donkey anti-rabbit, donkey anti-goat; all from Amersham or Jackson ImmunoResearch) for 1 h at room temperature. After washing 3–4 times in PBS, the sections were treated with avidin peroxidase (diluted 1:100 in 10 % FCS/PBS), and incubated for 1 h at room temperature. For visualization of the bound antibodies, diaminobenzidine (DAB) was used as chromogen.

**Table 2:** Antigen retrieval and primary antibodies

Antibody	Origin	Target	Dilution	Antigen retrieval	Source
AQP-1	Rabbit (pAB)	Aquaporin-1	1:500	0	Sc-20810; Santa Cruz, USA
AQP-4	Rabbit (pAB)	Aquaporin 4	1:250	0	Sigma-Aldrich, St. Louis, USA
C9neo	Rabbit (pAB)	Complement component C9	1:2,000	P	[39]
CD68	Mouse (mAB)	110-kD Transmembrane glycoprotein	1:100	St (E)	M0814; Dako, Glostrup, Denmark
GFAP	Rabbit (pAB)	Glial fibrillary acidic protein	1:3,000	St (E)	Z0334; Dako, Glostrup, Denmark
Ig	Sheep (pAB)	Human immunoglobulin	1:200	P	RPM1003; Amersham Pharmacia Biotech, Buckinghamshire, UK
MBP	Rabbit (pAB)	Myelin basic protein	1:2,500	0	A0623; Dako, Glostrup, Denmark
NF	Rabbit (pAB)	Neurofilament medium chain (150 kDa)	1:2,000	St (E)	AB1981; Chemicon, Temecula, CA, USA
PLP	Mouse (mAB)	Proteolipid protein	1:1,000	St (E)	MCA839G; Serotec, Düsseldorf, Germany
MAG	Mouse (mAB)	Myelin associated glycoprotein	1:1,000	St (E)	AB B11F7 [27]
TPPP/p25	Rabbit (pAB)	Tubulin polymerization promoting protein; Oligodendrocytes	1:250	St (E)	[15]
MOG	Mouse (mAB)	Myelin associated glycoprotein	1:1,000	St (C)	AB 8-18C5 [27]

*pAB* polyclonal antibody, *mAB* monoclonal antibody, *St* antigen retrieval in steamer, *E* EDTA buffer, *C* citrate buffer, *P* antigen retrieval with protease

For double staining, we combined two enzyme systems, peroxidase and alkaline phosphatase. After the single staining procedure using anti-mouse antibody, we further applied rabbit anti-human antibody and then incubated the sections with AP-conjugated anti-rabbit IgG and stained by Vector blue. Counter staining was done by hematoxylin or nuclear fast red, where applicable.

In human brain and spinal cord tissue astrocytes express besides AQP4 another water channel, i.e. aquaporin 1 (AQP1; Supplementary Figure 1; [48]). This is different from rodent CNS tissue, where AQP1 is exclusively expressed on the epithelial cells of the choroid plexus [24]. Since AQP1 is highly expressed in the human CNS in areas, destined to develop NMO lesions (Supplementary Figure 1) and is also present on the surface of astrocytes within NMO lesions, we used this protein as an additional marker to analyse the astrocyte reaction within the lesions.

### TUNEL staining

For the detection of cell death, we used the in situ cell death detection kit AP (Roche Applied Science, German). Briefly, dewaxed and rehydrated sections were covered by TUNEL reaction mixture for 60 min. After washing with PBS for 3 times, converter-AP was added on the slides for 30 min. After washing, the immunostaining was performed by Vector blue.

## Results

### Global pathology of active NMO lesions

**Fig. 1:**
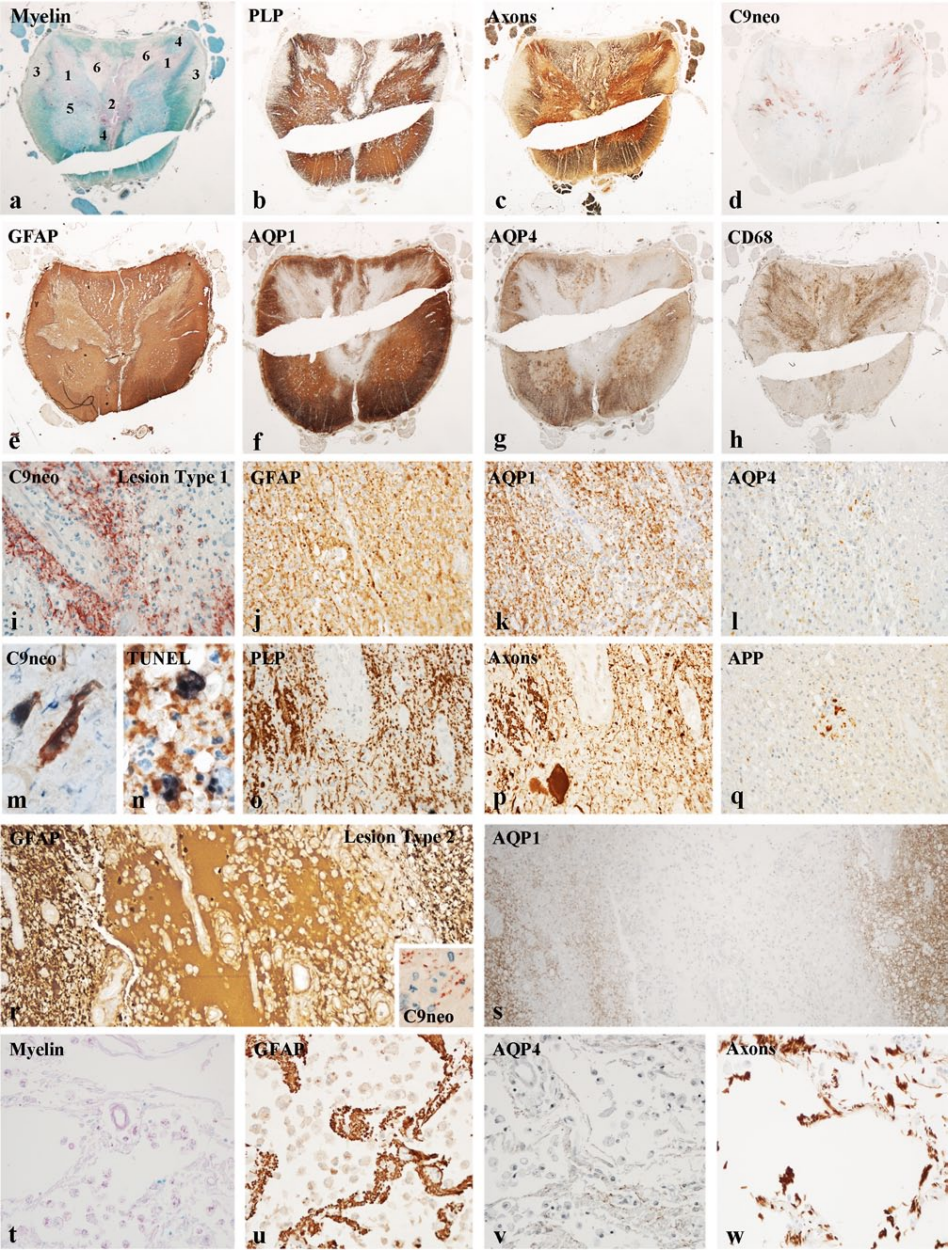
Different lesion types in the central nervous system of NMO patients: **a**–**h** distribution of different lesion types in the spinal cord of patients with neuromyelitis optica (patient 499, spinal cord). The different lesion types are characterized by different types of astrocyte pathology, demyelination, axonal injury, inflammation and complement C9neo deposition. The position of different lesion types, as characterized in detail in Figs. 1 and 2 are labeled by numbers in the sections stained for myelin (**a**); ×5. **i**–**q** Lesion type 1 is defined by extensive complement deposition and granulocyte infiltration (**i**); astrocytes are in part preserved and express AQP1, but have nearly completely lost AQP4 (**j**–**l**); degenerating astrocytes show C9neo reactivity on their surface (**m**) and fragmented TUNEL-positive DNA in the nucleus and dispersed in the cytoplasm, suggesting necrotic cell death (**n**); myelin (**o**) and axons (**p**) are partly preserved, but dystrophic swollen axonal spheroids are present (**p**), which have accumulated amyloid precursor protein (APP; **q**). **i**–**l** and **o**–**q** ×250; **m**, **n** ×1,100; **r**–**w** lesion type 2 reflects a destructive lesion with extensive loss of astrocytes (**r**, **s**), and liberation of GFAP rich exudates into the extracellular space (**r**); *insert* in **r** shows complement C9neo in macrophages; more advanced lesions **t**–**w** show cystic cavities with some macrophages and tissue bridges containing astrocytes (**u**), which have lost AQP4 (**v**) and axons (**w**), but being devoid of myelin myelin (**t**); **r**, **s** ×150; **t**–**w** ×250

Active lesions in both NMO and MS were defined by the presence of early CNS protein reactive degradation products in macrophages, as defined before in multiple sclerosis lesions [4]. In contrast to MS lesions, macrophages in active NMO lesions also contained GFAP reactive degradation products. All NMO cases included in this sample contained active lesions together with inactive destructive or demyelinated lesions at other sites of the central nervous system (Fig. 1). Active lesions in NMO showed profound inflammation. Inflammatory infiltrates were composed of T lymphocytes, B lymphocytes and macrophages. Profound microglia activation was seen in the periplaque white matter in both conditions. Inflammatory infiltrates were in part concentrated around vessels (perivascular cuffs) and in part dispersed throughout the lesion parenchyme. Abundant neutrophilic granulocytes and some eosinophils were present in a subset of active NMO lesions [28].

A more detailed inspection of sections with active NMO lesions, however, revealed that at least six different lesion types were present, suggesting different type- and stage-specific patterns of tissue injury (Table 3). They differed in the extent of complement deposition, granulocyte infiltration, acute astrocyte alterations, astrocyte loss, demyelination and axonal injury. They were found in the spinal cord and brain stem of all patients and also, when lesions were present at all, in the forebrain (Supplementary Figure 2). The different lesion types appeared in part side by side within the same tissue block (Fig. 1a–h), but their relative number in different brain regions of the same patient and of different patients varied (Supplementary Figure 2).

**Table 3:** Key pathological features of different lesions types in NMO

Les. Type	T	Gr	C9n	AG	Demy	OG loss	Ax loss
1	++	+++	+++	Necrosis	+	Apo	+
				AQP4 variable			
				AQP1 variable			
				GFAP variable			
2	+/++	±	±	AQP4 loss	Complete	+++	+++
				AQP1 loss			
				GFAP loss			
3	–	–	–	React. gliosis	+++	++	+++
4	±	–	–	AQP4 loss	–	–	–
				Clasmatodendrosis +			
5	++	–	–	Clasmatodendrosis +++	–	–	–
				AQP4 loss			
				AQP1 loss			
				GFAP loss			
**6**	++	–	–	Clasmatodendrosis +/++	Complete	+++	++
				AQP4 loss variable			
				AQP1 loss variable			
				GFAP loss ±			

*Les.Type* lesion type, *T* T cells, *Gr* granulocytes, *C9n* complement C9neo antigen, *AG* astroglia pathology, *Demy* demyelination, *OG* *loss* loss of oligodendrocytes, *Ax. Loss* axonal loss

±, minor or absent; +, minor; ++, moderate; +++, severe

#### Active NMO lesions associated with complement activation and granulocyte infiltration (lesion type 1)

The first lesion type was characterized by massive deposition of immunoglobulins and activated complement and high infiltration of the tissue by granulocytes (Fig. 1i). In such lesions astrocytes and their processes were covered in part with activated complement (Fig. 1m), and perivascular astrocyte processes were partly lost. The remaining GFAP positive astrocytes did not show AQP4 on their surface, but in part still expressed AQP1 (Fig. 1j–l). TUNEL staining showed DNA fragmentation in the nuclei of such astrocytes and TUNEL-positive DNA was also diffusely dispersed within the cytoplasm, a pattern characteristic for cell necrosis (Fig. 1n). Myelin and axons were relatively spared in comparison to astrocyte loss, but active demyelination and axonal injury was documented by apoptotic oligodendrocytes and by the presence of macrophages with early myelin degradation products together with acutely injured axons with accumulation of amyloid precursor protein and neurofilament reactivity in axonal spheroids (Fig. 1o–q). Analysis of different myelin antigens revealed little or no loss of myelin basic protein, proteolipid protein or myelin oligodendrocyte glycoprotein, while myelin associated glycoprotein was lost from most myelin sheaths [5] and oligodendrocytes were massively reduced in numbers within the lesions (Supplementary Figure 3).

#### Cystic lesions with extensive tissue destruction (lesion type 2)

In these areas most cellular components were lost and replaced by fluid filled cysts, which contained a variable number of macrophages and vessels with perivascular fibrosis (Fig. 1r–w). If such lesions still contained active areas of lesion type 1, C9neo antigen was present on astrocyte processes or in degradation products in macrophages at the lesion borders. Within the inactive center of such lesions some bridges of preserved tissue remained between the cystic cavities, which contained astrocytes and axons, but no myelin sheaths and only sparse oligodendrocytes (Supplementary Figure 3). AQP1 and AQP4 expression was largely lost within cystic lesions. When such lesions were present side by side with active lesions the cysts contained a protein-rich fluid, which was highly reactive for GFAP but not for aquaporins (Fig. 1r, s). This was no longer the case in more advanced destructive lesions (Fig. 1t–w).

#### Lesions resembling secondary Wallerian degeneration (lesion type 3)

**Fig. 2:**
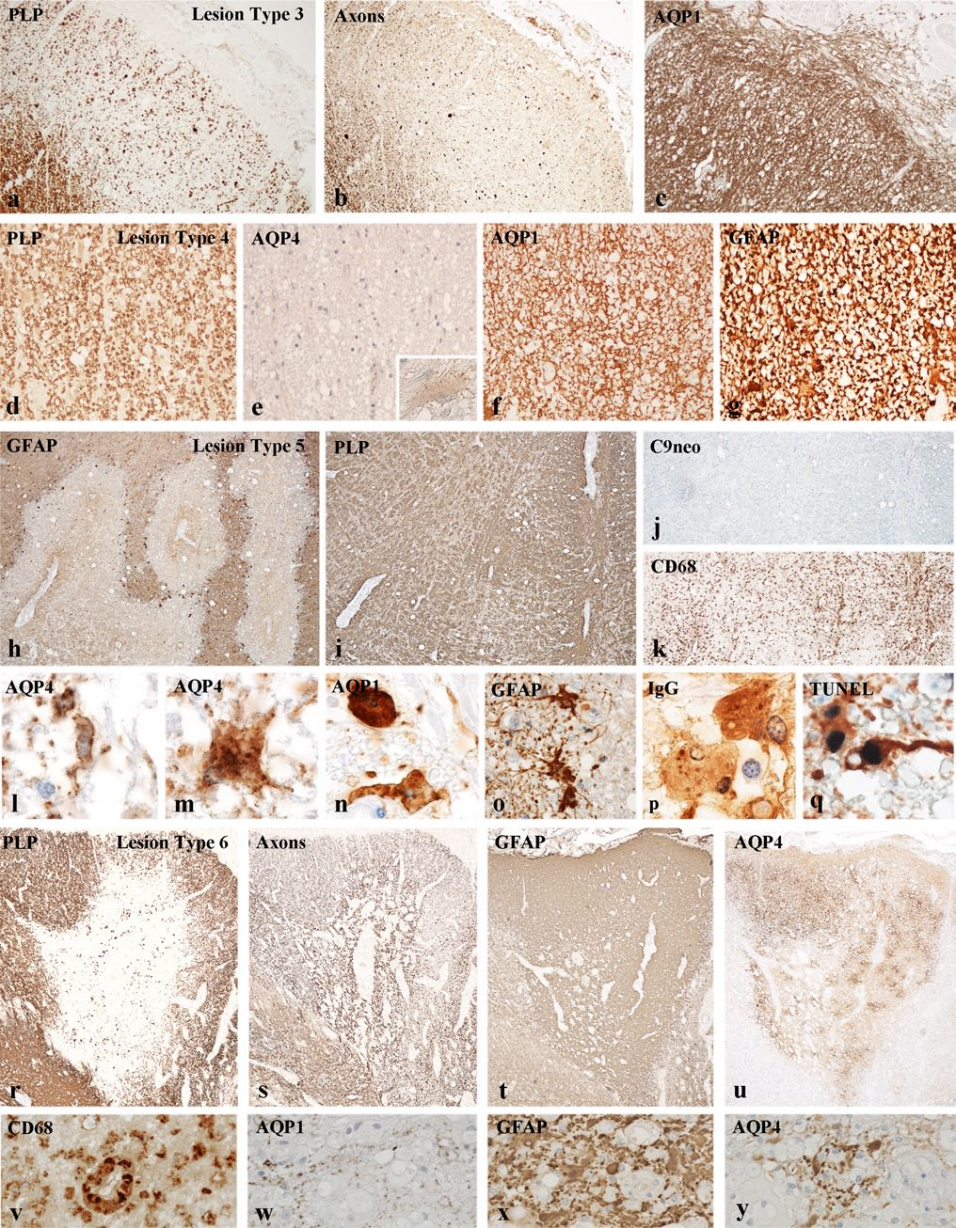
Different lesion types in the central nervous system of NMO patients [2]: **a**–**c** lesion type 3 represents areas of Wallerian degeneration; myelin sheaths (**a**) and axons (**b**) are lost in comparable degree; there is profound astrocytic gliosis (**c**); the astrocytes express GFAP and AQP1 and 4; (patient 499, spinal cord) ×30. **d**–**g** Lesion type 4 shows selective loss of AQP4 (**e**), while myelin sheaths (**d**), axons and neurons remain preserved; reactive astrocytes express GFAP (**g**) and AQP1 (**f**); some astrocytes also show granular intra-cytoplasmic reactivity for AQP4 (**e**, *insert*); (patient 499 spinal cord), ×250; *insert*: ×1,100. **h**–**q** lesion type 5 shows extensive loss of astrocytes (**h**) and astrocytic clasmatodendrosis (**l**–**q**), while myelin sheaths (**i**) and axons are preserved; there is profound macrophage infiltration (**k**), but no deposition of activated complement or granulocyte infiltration (**j**); astrocyte clasmatodendrosis is characterized by enlargement or peri-nuclear astrocyte cytoplasm and clumping and beading of cell processes (**l**–**q**), granular internalization of AQP1 (**n**), AQP4 (**l**, **m**) and IgG (**p**) and condensed nuclei (**m**, **n**, **p**) with DNA fragmentation (apoptosis; **q**); **h**–**k** (patient 216, medulla); ×50; **l**–**q** ×1,100. **r**–**y** Lesion type 6 is characterized by selective primary demyelination (**r**), axonal preservation (**s**), profound astrocytic gliosis (**t**) and a variable expression of AQP4 (**u**). AQP4 is lost in adjacent areas, which show features of complement-mediated active tissue destruction (adjacent lesion 1; Fig. 1a) or a destructive lesion pattern 2 (adjacent lesion 2, Fig. 1a); active demyelination is associated with macrophage infiltration (**v**); astrocytes show partial loss of AQP4 (**y**) and AQP1 (**w**) and some astrocytes with clasmatodendrosis (**x**, **y**); **r**–**u** (patient 499, spinal cord); ×20; **v**–**y** ×150

Such lesions were present in white matter tracts of the spinal cord. They showed an extensive loss of myelin, oligodendrocytes and axons and a profound fibrillary gliosis with densely packed GFAP and AQP1 reactive astrocytic processes, while loss of AQP4 was variable (Fig. 2a–c, Supplementary Figure 3).

#### Lesions with selective loss of AQP4 in the absence of other structural damage (lesion type 4)

This pattern of tissue injury was frequently seen in the vicinity of active NMO lesions. They were associated with mild to moderate perivascular inflammatory infiltrates and a variable degree of microglia activation, but no complement deposition or granulocyte infiltration. In sections stained for conventional cellular markers, myelin or axons, no pathological alterations were seen (Fig. 2d, Supplementary Figure 3). However, immunohistochemistry for AQP4 revealed profound or even complete loss of this protein on astrocytes, which still normally expressed AQP1 and GFAP. GFAP positive astrocytes either showed a normal structure and the presence of cell processes within the perivascular glia limitans. However, some of them were enlarged with increased perinuclear GFAP reactivity and contained intra-cytoplasmic granular AQP4 reactivity, suggesting endocytotic uptake (Fig. 2d–g) probably due to the AQP4Ab-induced internalization.

#### Active NMO lesions with astrocytic clasmatodendrosis (lesion type 5)

In these lesions, inflammatory infiltrates mainly consisted of T cells and macrophages. Reactivity for activated terminal complement on astrocytes and granulocyte infiltration were absent at the site of astrocyte injury. The characteristic hallmark of such lesions was the accumulation of bizarre astrocytes with massively enlarged peri-nuclear cytoplasm, presence of intra-cytoplasmic vacuoles and retraction, beading and clumping of the cell processes. AQP4 reactivity was partially lost from the surface of such astrocytes, but the cells contained abundant intracellular granules, reactive for AQP4, AQP1 and immunoglobulins. Many of these cells showed dense and condensed nuclei with DNA fragmentation, thus resembling apoptotic cells. Myelin sheaths, reactive for MBP, PLP and MAG, oligodendrocytes and axons were well preserved in the majority of these lesions, thus the presence of such lesions was not visible in sections stained for myelin (Fig. 2h–q, Supplementary Figure 3).

#### Lesions with astrocyte dystrophy and primary demyelination (lesion type 6)

These lesions were sharply demarcated areas of complete demyelination, loss of oligodendrocytes and axonal preservation (Fig. 2r–y, Supplementary Figure 3). Active demyelination was reflected by the presence of macrophages with early myelin degradation products. Also in these lesions prominent inflammation was present mainly consistent of T cells and macrophages. Granulocytes and deposition of activated complement were absent. GFAP positive astrocytes were present in the lesions in variable extent, and immunoreactivity for AQP1 and AQP4 showed more pronounced reduction compared to GFAP. Furthermore, astrocyte clasmatodendrosis, as described in lesion type 2, was seen in some of the remaining GFAP positive astrocytes.

#### Astrocyte pathology in multiple sclerosis lesions

**Fig. 3:**
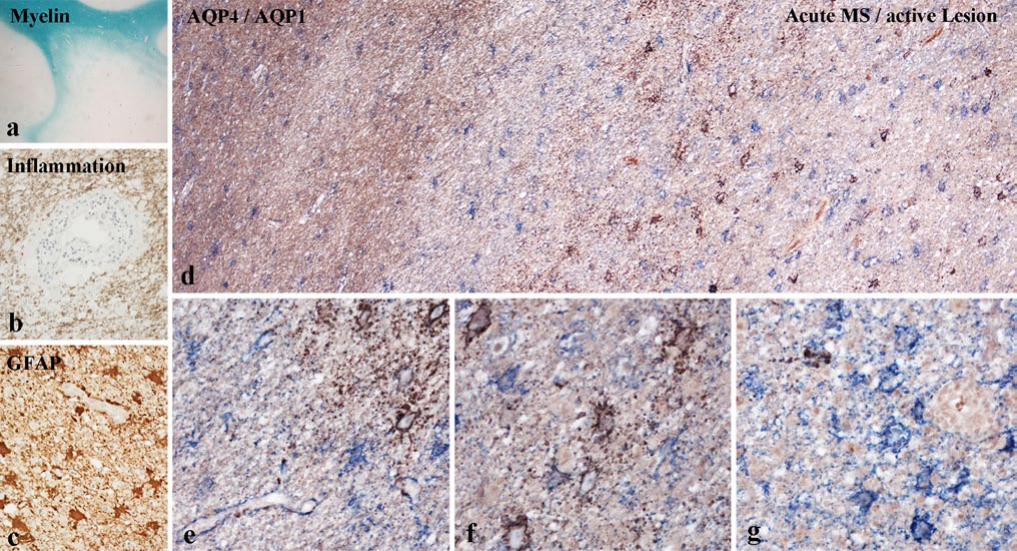
Astrocyte pathology in an aggressive active demyelinating lesion of acute MS: **a**–**c** large subcortical lesion with ill defined borders and some concentric banding of demyelination at the lesion edge (**a**) is associated with profound inflammation (**b**) and protoplasmatic astrocyte reaction (**c**). **a** ×2; **b**, **c** ×150. **d**–**g** Astrocyte pathology shown by double staining for AQP1 (*blue*) and AQP4 (*brown*); in the normal appearing white matter reactive astrocytes express AQP1 and AQP4 (**d**, *left*); in the active zone there is a concentric layering of AQP4 loss (*blue* cells) and adjacent zones with AQP1-reactive astrocytes that strongly express AQP4; **e**–**g** show higher magnifications of the areas shown in **d**. **d** ×300; **e**–**g** ×900

Active lesions dominated the pathology of acute MS (Fig. 3), although also a variable number of inactive lesions were present. In patients with progressive MS, classical active lesions were rare, while slowly expanding as well as inactive lesions were seen in variable numbers [21]. Active as well as slowly expanding lesions showed inflammation, mainly consistent of T cells and macrophages/microglia [7], but granulocytes and eosinophils were rarely seen. As described in detail before ([23], Fig. 3a–c) primary demyelination with oligodendrocyte apoptosis was the hallmark of initial tissue injury in active MS lesions. Furthermore, preferential loss of myelin-associated glycoprotein was prominent in active lesions of acute MS [27].

Concerning astrocytes, slowly expanding and inactive lesions in MS showed the MS-typical glial scar. Reactive astrocytes expressed GFAP, AQP4 and AQP1. However in a subset of fulminate active lesions in acute MS, loss of AQP4 was encountered, associated with the appearance of large protoplasmatic, sometimes multi-nucleated astrocytes, with retraction of perivascular astrocytic foot processes from the glia limitans (Fig. 3; [32, 33, 49]). These protoplasmatic astrocytes were intensely stained for GFAP and highly expressed AQP1. Furthermore, in immediately adjacent areas massive over-expression of AQP4 was seen around the cell body and proximal processes of some of the reactive astrocytes. Morphological signs of necrosis or apoptosis as well as evidence for DNA fragmentation in astrocytes were absent and no activated complement was detected on these cells (Fig. 3d–g).

## Discussion

The specific features in acute stages of NMO lesions are the loss of AQP4 [35, 36, 43] and the perivascular or subpial deposition of humoral factors such as immunoglobulin IgG and IgM, or activated complement (C9neo; [28, 43]). This apparently reflects a humoral immune attack against the glia limitans especially against the astrocyte foot processes by AQP4 reactive auto-antibodies. Based on these observations and experimental evidence it has been suggested that blockade of terminal complement activation, of Fc-receptor-mediated, antibody-dependent cellular cytotoxicity or of the recruitment of neutrophils and eosinophils should be followed as therapeutic strategies in NMO patients with active disease [42, 47, 53].

In this study, we show that tissue injury in the brain and spinal cord of NMO patients is complex and is mediated by at least two different mechanisms. In the first (lesion type I), active tissue injury is associated with antibody deposition and complement activation on astrocytes and their processes and this process is associated with profound recruitment of granulocytes and some eosinophils. This pattern can be well reproduced in in vivo rodent models, mediated by NMO antibodies in animals with autoimmune encephalomyelitis [3] as well as after local injection of NMO IgG into the mouse brain together with human complement [46]. Such lesions are in close topographical relation with inactive destructive lesions (lesion type 2) and we suggest that global overt tissue destruction and the formation of cystic cavities is the direct consequence of complement and granulocyte-induced tissue injury. In addition, the lack of astrocytes expressing the excitatory amino acid transporter (EAAT2) may promote excitotoxicity due to excess glutamate in the extracellular space [13, 30]. Interestingly, the cystic cavities in early stages of destructive lesions contain a protein-rich fluid, which is highly reactive for GFAP. Thus, liberation of soluble GFAP through necrotic destruction of astrocytes may be the reason for high GFAP levels in the patient’s cerebrospinal fluid during active phases of the disease [37, 51]. Furthermore, extensive axonal destruction in such necrotizing lesions will result in secondary Wallerian tract degeneration in rostral or caudal segments of the spinal cord (lesion type 3).

A second type of lesions (type 4 and 5) apparently developed in the absence of complement activation and granulocyte infiltration at the site of active tissue injury. This type of tissue injury affected astrocytes leaving other components of the nervous tissue unaffected. It was reflected either by selective loss of AQP4 on astrocytes (lesion type 4) or by profound astrocyte loss associated with degenerative astrocyte alterations at the lesion edge (lesion type 5).

As described before [35, 36, 43] astrocytes, which are still preserved around NMO lesions, may lose AQP4 from their surface. The functional significance of pure AQP4 loss is currently not clear. It has been suggested that such astrocytes are functionally impaired with respect to control of water homeostasis [12], although these in vitro results could not be confirmed by others [44]. In addition, in the human brain and in such NMO lesions, but not in the rodent CNS, astrocytes also express AQP1, which is similarly efficient in regulating water transport in comparison to AQP4 [14].

In lesions with astrocyte loss, the characteristic hallmark for tissue injury was loss of AQP4 and a pattern of astrocyte dystrophy, which bears similarities to astrocyte clasmatodendrosis. Clasmatodendrosis was initially described in the beginning of the 20th century, as a regressive morphological change of astrocytes with cytoplasmic swelling and vacuolation with beading and dissolution of their dendritic processes [16]. Recently, clasmatodendrosis has been recognized to be associated with various diseases such as vascular diseases including Binswanger’s leukoencephalopathy [10, 52] or epilepsy [19]. It appears to reflect a regressive change of astrocytes, which can be induced by several mechanisms, such as for instance energy deficiency [16, 19] or autophagic cell death [45], and which may finally result in astrocyte apoptosis [19]. In NMO this alteration of astrocytes seems to be directly induced by AQP4 antibodies in the absence of complement. This is supported by our finding that this type of astrocyte injury is associated with intra-cytoplasmic accumulation of AQP4, AQP1 and IgG-positive granules, apparently reflecting endosomal internalization of antibody-opsonized portions of the astrocyte cell membrane. Similar astrocyte changes have been described before in active NMO lesions [14]. We show further that many of the astrocytes with such alterations have shrunken, condensed and sometimes fragmented nuclei, which contain fragmented DNA. These changes are typical for apoptosis. Thus, there is apparently a pathway of astrocyte destruction in NMO lesions, which is different from complement and granulocyte mediated lysis. Interestingly, this disturbance and loss of astrocyte was not necessarily associated with demyelination nor neurodegeneration, which is in line with previous observations [41].

In addition we found lesions (lesion type 6) in NMO patients, characterized by complete demyelination and axonal preservation. Astrocytes within these lesions were present, but some showed changes of clasmatodendrosis, described above. It is likely that in these lesions, as in the destructive complement-mediated lesions, oligodendrocyte injury and demyelination follows astrocyte injury and loss. Interestingly, initial alterations of oligodendrocytes and myelin, consistent of oligodendrocyte apoptosis and loss of distal oligodendrocyte processes, which had originally been defined in a subset of active multiple sclerosis lesions [27], are also seen in actively demyelinating NMO lesions [5]. It has, thus, been suggested that in NMO as well as in MS, demyelination may follow an initial astrocyte injury [38]. Astrocytes are important for creating a homeostatic environment for oligodendrocytes. Energy demand of oligodendrocytes and axons is in part provided by astrocytes, transferring lactate through gap junctions to oligodendrocytes [9]. Loss of gap junction-related connexins that are expressed on astrocytes or oligodendrocytes has been found in active lesions of MS and NMO [34, 49]. In addition, astrocytes express the excitatory amino acid transporter 2 (EAAT2), which removes toxic glutamate from the extracellular space and can, thus, reduce excitotoxic cell death of neurons and oligodendrocytes [30]. Since demyelination in a subset of MS lesions is associated with oxidative damage, mitochondrial injury [29] and a state of virtual hypoxia [1], it could also be that such lesions are induced in both conditions by additional hypoxia-like mechanisms. In NMO, severe tissue edema may impair blood microcirculation. In addition, oxidative damage and hypoxia-like tissue injury can be amplified by glutamate toxicity, which is expected to occur in such NMO lesions due to the loss of EAAT2 [13, 30].

Although loss of AQP4 has been described before in actively demyelinating lesions in a subset of MS patients [32, 33, 49] and was also seen in this study, this was not associated with complement activation, granulocyte infiltration, astrocyte destruction or loss [35, 43] or astrocyte clasmatodendrosis. AQP4 loss in this condition rather reflected retraction of perivascular astrocyte processes, similar to that present in CNS lesions induced by severe innate immunity-driven inflammation [49]. The lack of astrocyte loss or complement deposition does not support the view that antibodies against Kir 4.1, which have recently been described in around 47 % of all MS patients [50], destroy astrocytes in the lesions in a complement-dependent manner.

For clinicians in front of NMO patients, these various patterns must be informative. For Type 1 and 2, we should consider the earliest strategy to remove the humoral factors such as immunoglobulin and complement in the acute stage, probably by plasmapheresis [55], and to decrease complement activity possibly by anti-C5 antibodies [40], by preventing granulocyte infiltration [42, 47]. Alternatively, blocking endogenous auto-antibody binding with aquaporumab, which binds to AQP4 but is unable to activate complement or interact with effector cells [53] may be beneficial. Inhibition of complement activation or granulocyte infiltration are promising to at least partially block lesion types 1–3, and thus to ameliorate acute disease exacerbations. However, it is unlikely that these treatment strategies have a major effect on the lesion types 4–6, and therefore will not fully prevent disease progression. In contrast, persistent reduction of serum auto-antibody titers, for instance by immunosuppressive drugs or anti-CD20 antibodies [17], or blockade of AQP4 antibody binding by small molecule inhibitors [54], may be more effective against the entire spectrum of NMO lesions. Finally, the presence of destructive spinal cord lesions and the resulting Wallerian tract degeneration may explain, why in NMO patients, after the improvement of motor functions of transverse myelopathy, spastic paraparesis and pain with severe girdle sensation tend to persist chronically [18]. Finally, our current results provide further evidence that NMO is a disease, which is distinctly different from MS, and that astrocyte injury and destruction is the primary event, which occurs in active lesions. However, the mechanisms of tissue injury are complex and effective neuroprotective therapy may have to target several different mechanisms in parallel.

## Electronic supplementary material

Below is the link to the electronic supplementary material.

Supplementary material 1 (DOCX 23 kb)

Supplementary material 2 (TIFF 5901 kb)

Supplementary material 3 (TIFF 1619 kb)

Supplementary material 4 (TIFF 16372 kb)

Supplementary material 5 (TIFF 12176 kb)

## Abbreviations

AQP1: Aquaporin 1
AQP4: Aquaporin 4
GFAP: Glial fibrillary acidic protein
NMO: Neuromyelitis optica
MS: Multiple sclerosis
EAAT2: Excitatory amino acid transporter 2
CNS: Central nervous system
PBS: Phosphate buffered saline
FCS: Fetal calf serum
