# Supplementary material for: Presence of six different lesion types suggests diverse mechanisms of tissue injury in neuromyelitis optica
Source: Acta Neuropathol. 2013 Apr 12;125(6):815–27. doi: 10.1007/s00401-013-1116-7 (PMC3661909; doi:10.1007/s00401-013-1116-7)
Supplement: Supplementary file 1 — Supplementary material 1 (DOCX 23 kb) [file 401_2013_1116_MOESM1_ESM.docx]

Supplementary Figure 1a: Expression of AQP1 and AQP4 in the human cortex, optic chiasm and medulla oblongata:

AQP1 is mainly expressed in the white matter, while AQP4 is present in grey and white matter.

Supplementary Figure 1b: Western blot of AQP1 and AQP4 expression in the human and rat white matter:

AQP4 is expressed in human and rat white matter, while AQP1 is seen only in human samples.

Supplementary Figure 2: Distribution of different lesion types in different tissue samples of different NMO patients (the numbers represent the location of the different lesion types)

a-c: Thoracic spinal cord of patient 499, shown in Figure 1a-h; a: AQP4; b: AQP1; c: GFAP;

d-e: Medulla oblongata of patient 216 shown in Figure 2h-q; d: GFAP; e: PLP;

f: Spinal cord of patient 2001-50 with active NMO lesions; immunohistochemistry for C9neo antigen;

g-i: Medulla oblongata (g) and spinal cord (h,i) of patient 12193 with active NMO lesions; g: luxol fast blue myelin stain; h: GFAP, i: double staining for AQP1 (brown) and AQP4 (blue).

j-k: Brain sections from patient 499 with active and inactive NMO lesions; luxol fast blue myelin stain; x 1.2

Supplementary Figure 3: Myelin and oligodendrocyte pathology in different NMO lesions.

a-c: Spinal cord lesion from patient 499, as shown in Figure 1. There is complete demyelination in lesion type 6, while myelin is partly preserved in the adjacent lesion type 1 (a: PLP). Immunohistochemistry for myelin associated glycoprotein (MAG) shows loss of MAG in both lesions; a and b: x 20; c: x 60;

d-i: TPPP/p25-positive oligodendrocytes in the normal appearing white matter and in different lesions types in the spinal cord of patient 499. Insert in (e) shows TUNEL staining (blue) in one of the oligodendrocytes (brown). The same lesions which are shown in Figures 1 and 2 have been selected for documentation of oligodendrocyte density.

j-l: TPPP/p25-positive oligodendrocytes in the normal appearing white matter and in lesions in the medulla oblongata of patient 216.

The images show in comparison to the normal appearing white matter a massice reduction of oligodendrocyte density in lesion types 1, 2 and 6, a moderate reduction in lesion type 3 and oligodendrocyte density similar to that in normal appearing white matter in lesion types 4 and 5.

Supplementary Material and Methods:

Western Blot

Lewis rats were housed in the Decentral Facilities of the Institute for Biomedical Research (Medical University of Vienna) under standardized conditions. An 8-week old animal was killed with CO_2_ and perfused with 120 ml phosphate buffered saline (PBS). Aliquots of 35 mg dissected white matter were snap-frozen in liquid nitrogen and stored at -80 °C.

The normal human white matter sample (frontal white matter, not containing choroid plexus; also 35 mg) was derived from the Clinical Institute of Neurology (Medical University of Vienna) and stored at -80 °C.

Tissue samples were homogenized in ice-cold Ripa buffer (50 mM Tris/HCl (pH 7.5), 150 mM NaCl, 0.1% SDS, 0.5% sodium deoxycholate, 1% NP-40; supplemented with complete Mini Protease Inhibitor cocktail tablet (cat# 04693124001; Roche Diagnostics GmbH, Mannheim, Germany) prior to usage) using a Potter-Elvehjem tissue grinder. Thereafter, the homogenate was passed 10 times through a 27G needle attached to a 1 ml syringe, incubated on ice for 20 min and centrifuged at 16000xg for 20 min in a cooled microcentrifuge. Protein concentration of the samples was determined measuring absorbance at 280 nm using a NanoDrop 2000 Spectrophotometer (PeqLab, Erlangen, Germany). NuPAGE® LDS sample buffer and NuPAGE® Reducing Agent (cat# NP0007 and NP0009, respectively; both Invitrogen) were added and samples were heated at 70 °C for 10 min. Equal concentrations of the samples were loaded onto 10% SDS gels and transferred on Immobilon-P transfer membranes (Millipore). Subsequently, membranes were blocked in blocking buffer (1.5% milk powder, 0.1% Tween in PBS) for 1 h at room temperature. Blots were probed for AQP1 (rabbit anti-human; 1:500; cat# sc-20810; Santa Cruz Biotechnology Inc.), AQP4 (rabbit anti-rat; 1:800; cat# A5971; Sigma-Aldrich) or beta-Actin (mouse anti-human/rat; 1:20000; cat# ab6276; abcam) overnight at 4 °C. Cross-reactivity of AQP1 and AQP4 antibodies for rat and human tissue, respectively, had been confirmed before. The next day, the blots were washed 3 times for 10 min in fresh blocking buffer. Alkaline phosphatase-conjugated secondary antibody incubations (anti-rabbit IgG (1:2000; cat# 111-056-045) and anti-mouse IgG (1:2000; cat# 115-055-072); both Jackson ImmunoResearch Laboratories) were done for 1 h at room temperature. After another 3 washing steps, the membranes were exposed to a chemiluminescent substrate (CDP-Star® (cat# T2305; Applied Biosystems) in assay buffer (1 mM MgCl_2_, 1% diethanolamine in distilled H_2_0, pH 10)). Subsequent imaging was done using the Molecular Imager® ChemiDoc^TM^ XRS System (Bio Rad Laboratories). Images were further processed with Image Lab version 4.0 (Bio Rad Laboratories).
