# Supplementary figures and images for: Presence of six different lesion types suggests diverse mechanisms of tissue injury in neuromyelitis optica
Source: Acta Neuropathol. 2013 Apr 12;125(6):815–27. doi: 10.1007/s00401-013-1116-7 (PMC3661909; doi:10.1007/s00401-013-1116-7)

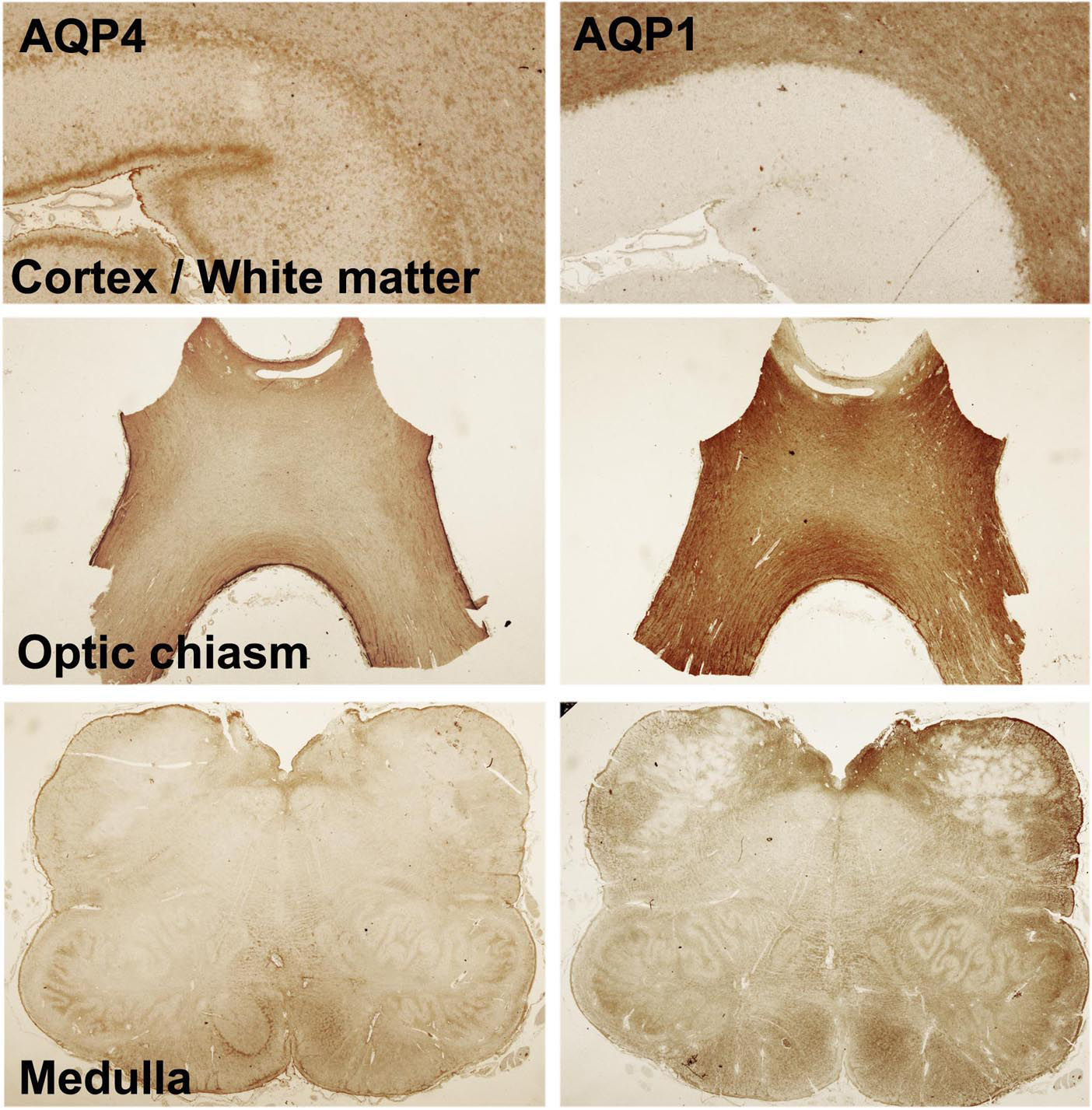

Supplement: Supplementary file 2 — Supplementary material 2 (TIFF 5901 kb) [file 401_2013_1116_MOESM2_ESM.tif]

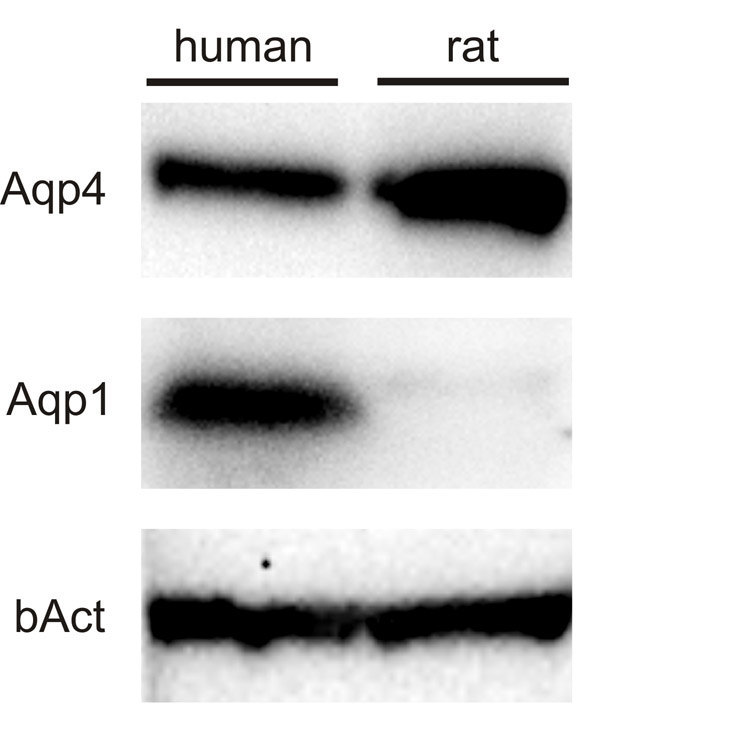

Supplement: Supplementary file 3 — Supplementary material 3 (TIFF 1619 kb) [file 401_2013_1116_MOESM3_ESM.tif]

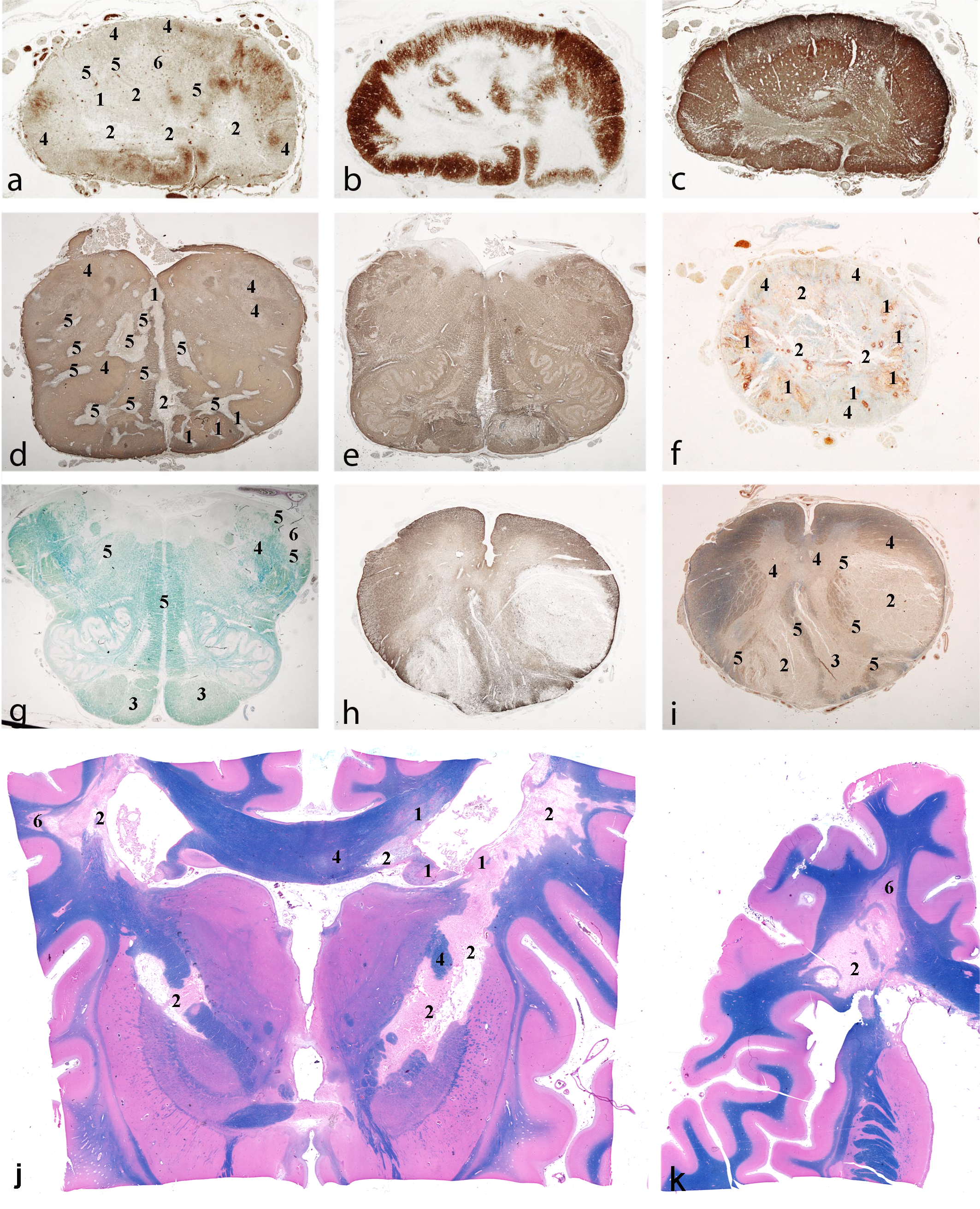

Supplement: Supplementary file 4 — Supplementary material 4 (TIFF 16372 kb) [file 401_2013_1116_MOESM4_ESM.tif]

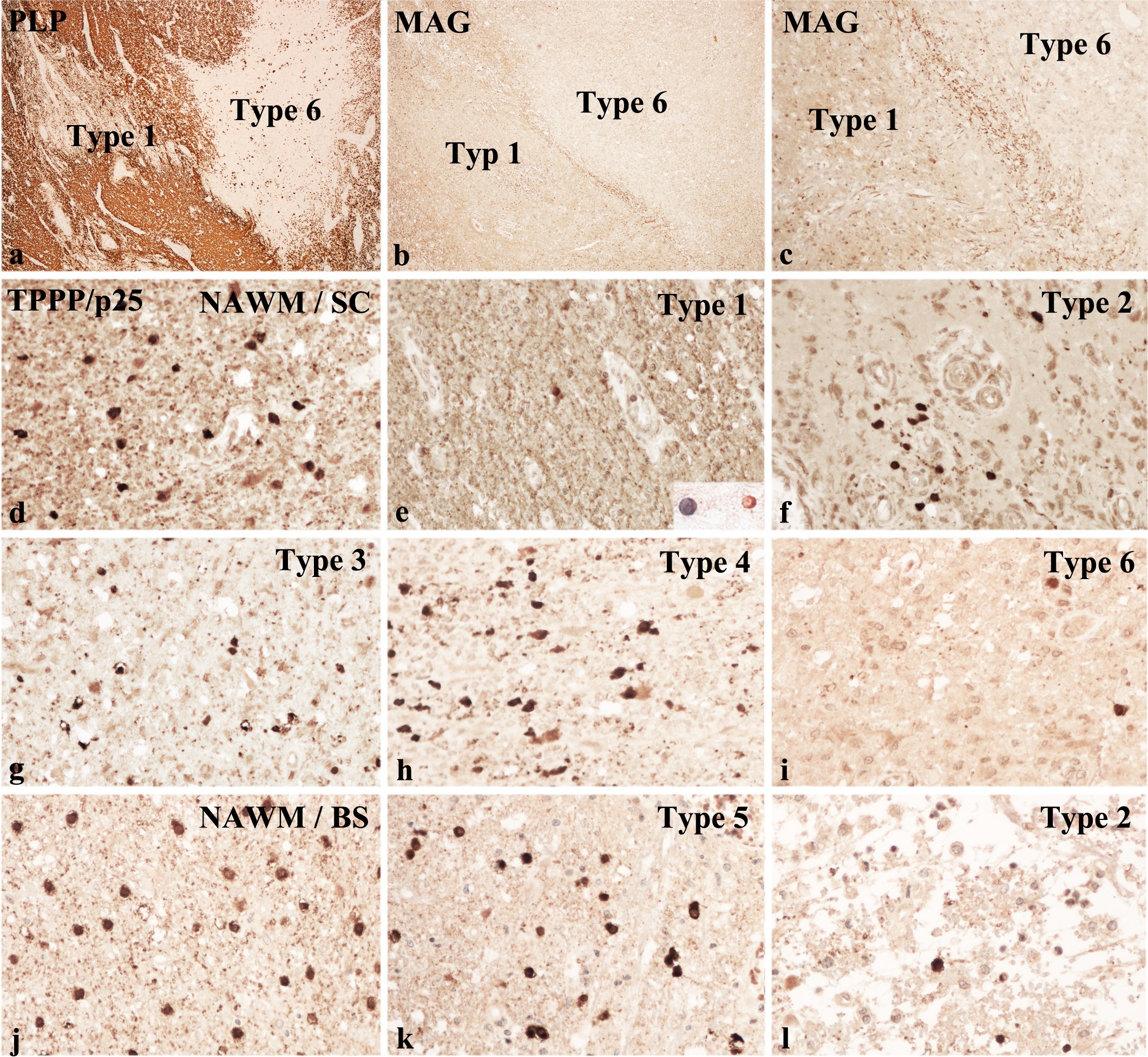

Supplement: Supplementary file 5 — Supplementary material 5 (TIFF 12176 kb) [file 401_2013_1116_MOESM5_ESM.tif]
